# Too Close for Comfort: Stigma by Association in Family Members Who Live with Relatives with Mental Illness

**DOI:** 10.3390/ijerph20065209

**Published:** 2023-03-16

**Authors:** Joel O. Goldberg, Suzanne A. McKeag, Alison L. Rose, Heather Lumsden-Ruegg, Gordon L. Flett

**Affiliations:** Department of Psychology, York University, Toronto, ON M3J 1P3, Canada

**Keywords:** mental health stigma, social isolation, mattering, loneliness, stigma by association

## Abstract

Stigma by association is described in qualitative research of family members who have relatives diagnosed with mental illness, depicting their sense of public shame for having these relationship ties. However, there have been relatively few empirical studies thus far, in part due to the isolation of family members affecting research recruitment. In order to address this gap, an online survey was administered to 124 family members, comparing those who live in the same home with their ill relative (*n* = 81) and those who do not (*n* = 43). A remarkable incidence of one in three family members reported experiencing stigma by association. Those living with an ill relative reported comparatively higher levels of stigma by association using an adapted questionnaire measure. Both groups experienced loneliness (moderate levels), but importantly, the cohabiting relatives perceived themselves as lacking support from friends and other family members. Correlational analyses revealed that those with heightened stigma by association reported heightened anti-mattering: that is, feeling that other people treat them as if they are insignificant and invisible. Anti-mattering was also associated with more loneliness and reduced social support. Our discussion focuses on the theme that family members who actually live with mentally ill relatives experience heightened social isolation that is under-recognized due to public stigma concerns, compounded by feeling their own lives do not matter to others. Public health implications are considered for the stigmatized family members who appear to be particularly marginalized.

## 1. Introduction

The COVID-19 pandemic with societally prescribed social restrictions [1] has served to sharpen the focus on the public health consequences of social isolation and loneliness. Previous meta-analytic work [2] has highlighted the impact of both perceived and actual social isolation on early mortality. However, within our broad society, it is individuals with serious mental illness (SMI) and their families who are often most isolated and who report a profound sense of social exclusion related to perceived stigmatization [3]. Family members who have a relative with SMI often assume demanding caregiving responsibilities that can become quite distressing [4] and place them at risk for burnout [5]. A recent mixed-methods study of family caregivers of individuals with SMI [6] poignantly depicted the extreme social isolation of these family members who were found to have exceedingly small social networks, an isolation which the authors ascribed to stigmatization. Specifically, family members reported the need to be “secretive about the (relative’s) mental illness… so they reduce their social interactions”. This is consistent with earlier work describing how family members feel avoided by friends, relatives, and other people in their communities. They hide their relationship with their mentally ill relative to evade stigma [7], and they believe that most people hold negative views of relatives of people with mental illnesses [8].

Corrigan and Miller [9] described how family members can experience a sense of shame, blame and contamination in their perceived stigma of being associated with their relative with SMI. This kind of stigma has been previously referred to as ‘courtesy’ stigma or ‘family’ stigma [9] but is now typically termed stigma by association [10]. Research studies of stigma by association using qualitative methods were conducted by van der Sanden et al. [11,12] in the Netherlands with Dutch family members of people with mental illness. Through interviews with family members, the authors found that 74% had experienced stigma by association [11]. Dominant themes reported included family members being blamed for the mental illness of their relative and experiencing social exclusion, which resulted both from friends, relatives, and other community members pulling away from participants and from participants withdrawing from people to avoid stigma. Additional qualitative research attests to the existence and importance of stigma by association among family members (see [13,14]).

What is unclear at present is whether family members who are actually living with versus living apart from their relative with SMI experience more stigma, more loneliness, and more isolation. Research findings thus far are mixed and are mainly restricted to qualitative reports. This is likely due, in part, to the difficulties in participant recruitment. Some research has suggested that there is a similar level of caregiver burden independent of joint household status [15,16]. Others [10] suggest more clearly that family members who actually live with their ill relatives experience a heightened caregiver burden. However, there remains a gap in the literature regarding the impact and consequences of loneliness and social isolation and stigma by association in this vulnerable group. There is a paucity of empirical studies that have been conducted in the area, which thus far has mainly been restricted to qualitative research.

The current study had two primary goals. First, we compared family members who are either living with or not cohabiting with their relative with serious mental illness to determine their relative levels of experienced stigma by association, loneliness, social support, and feelings of not mattering. Second, in the sample of family members as a whole, we examined the correlates of feelings of not mattering. We hypothesized that feelings of not mattering would be associated with elevated levels of loneliness and lower levels of social support, in accordance with past findings with young adults and other groups (see [17,18,19,20]). In addition, however, we wished to explore how feelings of not mattering might be linked with the experience of stigma by association. A link between feeling insignificant and stigma by association would be in keeping with the notion that being made to feel insignificant and invisible contributes to a more general sense of being devalued, judged, and stigmatized by others. This is thought to be felt most acutely among family members with a relative with a mental illness.

## 2. Methods

### 2.1. Participants

Participants were recruited through organizations that support family members who have relatives with mental illnesses. The Canadian Mental Health Association, the Institute for Advancements in Mental Health (formerly the Schizophrenia Society of Ontario), and Reconnect Community Health Services sent recruitment email advertisements communicating the opportunity to take part in the study. Clients who were engaged in their programs and services and had consented to receive follow-up correspondence were contacted. Additional participants were recruited through the Schizophrenia Society of York University (SSY), which promotes schizophrenia awareness and stigma reduction. The SSY posted the advertisement communicating the opportunity to take part in the study on their social media platforms (e.g., Instagram). Participants received a $5.00 (CND) coffee shop gift card as compensation for completing the study. The participants accessed the survey through the Qualtrics XM platform via an online link.

Data were collected from 254 individuals who provided informed consent and self-identified as relatives of people with serious mental illnesses, including but not limited to psychotic disorders (e.g., schizophrenia) and affective disorders (e.g., bipolar), and excluding neurocognitive disorders (e.g., dementia). Cases were removed from the data set for participants who had invalid profiles (*n* = 8) and missing data (*n* = 122). The final sample consisted of 124 relatives of people with mental illness, who spent an average of 45.3 (*SD* = 36.4) minutes online responding to the survey.

Participants’ demographics are noted in Table 1. The family members were divided into two groups. One group was comprised of people who cohabited with their relative with mental illness (*n* = 81), henceforth referred to as the cohabiting group, while the other group was comprised of people who did not live with their relative with mental illness (*n* = 43), henceforth referred to as the non-cohabiting group. Individuals in the cohabiting group ranged from 18 to 80 years old, with a mean of 44.0 years old (*SD* = 17.1). Participants in the non-cohabiting group ranged from 20 to 80 years old, with a mean of 53.8 years old (*SD* = 17.7). The cohabiting group was significantly younger overall than the non-cohabiting group (*t*(*df* = 78.40) = −2.91, *p* < 0.01). Inspection of additional demographics showed generally comparable patterns of distributions between the groups, with the most notable differences being that those in the non-cohabiting group self-identified more as “mother/father” (44%; *n* = 36 versus 58%; *n* = 25), less as spouse/partner (11%; *n* = 14 versus 0%; *n* = 0), and more as “other” (2%; *n* = 2 versus 19%; *n* = 8).

The demographics for the participants’ relatives with SMI are noted in Table 2. The age of the relatives with SMI was not significantly different between the groups. Consistent with participant demographics, an inspection of additional demographics of the relatives with mental illness generally showed comparable distributions between the groups. Importantly, clinical diagnoses of relatives with mental illness were relatively evenly distributed between the groups. Each participant could report more than one mental health condition. This corresponds to concurrent disorders often experienced by relatives with SMI and aligns with previous research which describes the characteristics of relatives with SMI [11].

### 2.2. Measures

Demographics were collected through a questionnaire that asked participants to report the age, gender, and race of themselves and their relative with SMI, their relationship to the relative with SMI, and their understanding of the mental illness(es) that their relative is experiencing.

- Stigma by Association Scale (SAS; adapted from Tessler & Gamach [21])

Stigma by association was measured using a 9-item SAS questionnaire originally published by Tessler and Gamach [21] as a subscale of their toolkit. We adapted the measure to specify its relevance for any family member of a person with an SMI (see Supplementary Materials). Therefore, items in the current measure substituted the term “relative” in lieu of the more general term “(NAME)” [21]. Further, minor edits were made to make items more concise and to keep the terminology about their “relative’s mental illness” consistent throughout the measure. An example item from the SAS is: “I have felt the need to hide my relative’s mental illness”, with participants selecting one of five options on a Likert scale ranging from “Never” to “Always”, scoring 1–5. The range of possible scores on the SAS is 9–45, with higher scores indicative of more stigma. A cutoff score of 27 (or higher) was established to identify those who report experiencing stigma by association. Cronbach’s alpha was very good for the SAS at 0.88.

- Multidimensional Scale of Perceived Social Support (MSPSS; [22])

Perceived social support was measured using the three subscales of the 12-item MSPSS (MSPSS; [22]), which assesses support from a significant other, support from family, and support from friends. Participants were asked to indicate how they felt about a series of statements by selecting one of seven options on a Likert scale ranging from “very strongly disagree” to “very strongly agree”. Sample items include the statement: “There is a special person who is around when I am in need” from the support from significant others subscale, “My family really tries to help me” from the support from family subscale, and “I can count on my friends when things go wrong” from the support from friends subscale. The range of possible scores on the MSPSS is 1–7; higher scores are associated with more social support. Cronbach’s alpha was found to be very good for the MSPSS subscales, at 0.87 for the significant others scale, 0.91 for the support from family scale, and 0.91 for the support from friends scale.

- General Mattering Scale (GMS; [23])

The GMS [23] was administered to measure how much participants felt that they generally mattered to other people. Participants were asked to select one of four options, from “1 = Not at all” to “4 = A lot”, in response to five statements, such as “How important do you feel you are to other people?” Scores on the GMS [23] can range from 5 to 20, with higher scores representing a greater sense of generally mattering to other people. Cronbach’s alpha was found to be lower than usual (see [24]) but acceptable for the GMS at 0.66.

- Anti-Mattering Scale (AMS; [18])

Anti-mattering pertains to the feeling that others are treating the individual as unimportant and insignificant [18]. Participants were asked to select one of four options, from “1 = Not at all” to “4 = A lot”, in response to five statements, such as “How often have you been made to feel by someone that they don’t care about what you think or what you have to say?”. AMS scores can range from 5 to 20, with higher scores representing a greater sense of being made to feel unimportant and invisible to other people. Cronbach’s alpha was found to be very good for the AMS at 0.87.

- UCLA Loneliness Scale (UCLA LS; [25])

The UCLA Loneliness Scale [25] was used to evaluate how frequently participants felt lonely. Participants were presented with 20 statements, such as “I have nobody to talk to”, and instructed to choose one of four responses, from “1 = I never feel this way” to “4 = I often feel this way”. Scores on the UCLA Loneliness Scale [25] can range from 20 to 80, and higher scores are associated with feeling lonely more often. Cronbach’s alpha was excellent for the UCLA Loneliness Scale [25] at 0.95.

Finally, participants were asked to provide a short answer to the question, “Please describe any experiences you have had with stigma as the family member of a person with mental illness.”

### 2.3. Procedure

Participants gave their informed consent and responded to the survey online. They were administered the demographics questionnaire, the five questionnaire measures, and the short answer question noted above. In addition, participants answered other questionnaire measures and short answer questions as part of a larger ongoing program of research.

### 2.4. Statistical Analyses

Quantitative data were analyzed in R Studio [26]. Welch’s independent *t*-tests were computed to assess differences between the cohabiting group versus the group of people who did not cohabit with their relative with mental illness on the SAS (adapted from Tessler & Gamach [21]), GMS [23], AMS [18], MSPSS subscales [22] and UCLA Loneliness Scale [25]. Correlations were computed between the dependent measures and the AMS [18].

For the qualitative analyses, keyword themes were identified in the responses in accordance with the qualitative analysis stage-by-stage process [27], with the exception that we did not have an opportunity to include the ‘member checking’ stage since we were unable to return to reach out to the participants.

## 3. Results

Means and standard deviations for the cohabiting group and non-cohabiting group on the variables examined are noted in Table 3.

The prevalence of self-reported stigma by association in the community sample was determined to be quite high (*n* = 43) at a remarkable one in every three family members, based on the SAS cutoff score. It may even be higher in the general population since our sample was recruited through community organization connections. A key finding, based on the SAS (adapted from Tessler & Gamach [21]) questionnaire, was that participants who live with a relative with SMI report significantly higher levels of stigma by association (*t*(67.06) = 3.02, *p* < 0.003; *d* = 0.62). The experience of stigma by association was poignantly described in the qualitative response of a 58-year-old mother who resides with her 24-year-old son with SMI: “Raising a child has been very difficult due to stigma. Being blamed as a ‘bad parent’ was a frequent occurrence for years; from immediate family to strangers, to teachers, to health professionals. It was excruciatingly difficult, and contributed to chronic feelings of self-blame, feeling like a failure, feelings of helplessness, hopelessness, confusion, chaos, isolation…”. Her vivid depiction of many painful emotions tied to stigma was a theme described by other participants and clearly compounded by social isolation and rejection (“my sister has cut me off since discovering my family member’s illness”).

Analyses of the subscales of the Multidimensional Scale of Perceived Social Support (MSPSS; [22]) confirmed a remarkably consistent pattern in which the cohabiting family member group perceived themselves as having significantly less support from friends (*t*(92.17) = −3.03, *p* < 0.003; *d* = 0.56), family (*t*(89.37) = −2.56, *p* < 0.011; *d* = 0.48), and significant others (*t*(84.89) = −3.16, *p* = 0.002; *d* = 0.60). A 62-year-old mother who resides with her 32-year-old son, diagnosed with schizophrenia, shared a powerful demonstration of her lived experience of social isolation and its relationship to stigma: “A conversation happened with my son telling family that he had just gotten out of hospital and was diagnosed with schizophrenia. The long and short of it being my family has nothing to do with us now, we are avoided… I told family and they shut us out, I am so hurt and angry at their hypocrisy!”

Analysis of the General Mattering Scale (GMS; [23]) revealed a trend in which people who live with their relative with SMI experience a lower sense that their lives truly matter compared with family members who do not live with their relative (*t*(75.72) = −1.75, *p* = 0.08; *d* = 0.35). This sentiment was reflected by a 24-year-old daughter who resides with her father with SMI, who indicated that her experience of stigma involved “Not being able to talk about my own experiences because it is not as important as the individual with the mental illness.” There were no group differences in anti-mattering (*t*(69.62) = 1.14, *p* = 0.26; *d* = 0.22).

Family members who reported experiencing stigma by association (based on the SAS cutoff score) were found to be experiencing moderate levels of loneliness (*M* = 50.5; *SD* = 12.7). However, mean scores did not differ significantly between the groups on the UCLA Loneliness Scale [25] (*t*(87.11) = 1.16, *p* = 0.25).

Correlations among the measures for the entire sample (*n* = 124 except for a very few instances of missing data) can be found in Table 4.

What was clear among all participants were findings that heightened stigma by association was linked to the strong sense that others treat them like they are insignificant or not truly valued, based on the AMS correlation (*r* = 0.41, *p* < 0.001). Further, there was a significant pattern of correlations between anti-mattering (AMS) and the variables of significant other (SO), family member (FM) and friend (FR) social supports (MSPSS), as well as between anti-mattering (AMS) and loneliness (UCLA Loneliness), such that those with reduced social supports and those with heightened loneliness report a significantly reduced sense that their lives matter to others. Further, heightened loneliness (UCLA Loneliness) was strongly associated with all aspects of lack of social support: that is, feeling unsupported by family members, significant others, and friends.

## 4. Discussion

The study found a startling one in three family members with relatives with SMI reported experiencing stigma by association. These family members were found to be experiencing, on average, at least moderate levels of loneliness in what is, to our knowledge, among the very first empirical studies of loneliness, social isolation, and stigma by association in a North American family member sample. The findings are consistent with recent research that found so-called ‘loneliness in the presence of others’ in family members who care for a relative with severe mental illness in Iran [6] and are also consistent with past research in Scandinavian family members [28,29]. Our findings also echo prior qualitative research that found family members of people with SMI experienced stigma by association and perceived themselves as lacking social support [7,30], particularly those who cohabited with their relatives [31].

Specifically, family members who live with a relative with SMI were found to feel significantly more social isolation compared with those family members who do not cohabit with their relative with SMI, according to family, friends, and significant other social support measures. The extent of their isolation is considerable, reflected in the striking findings that their MSPSS subscale levels were considerably lower than the original MSPSS psychometric validation studies [32] and much lower than those obtained recently from a community maternal caregiver sample [33]. The public health implications of this finding are clear, according to a study conducted in Turkey on social support in family caregivers [5]: that is, family caregivers with reduced social support are at heightened risk for burnout. Individuals who experience psychosis are themselves among the loneliest and socially isolated of adults, according to national surveys conducted in Australia [34]. The current research suggests that family members who live with relatives with SMI may also be quite marginalized in society, related to their experiences of stigma by association.

However, the findings of the current study point to the importance of ‘mattering’ as a key factor in the experience of lack of social support experienced by family members. Specifically, family members who are experiencing the most loneliness and who feel the most socially isolated are missing the sense that their lives truly matter to others; they are experiencing what has been termed a *double jeopardy* of feeling both lonely and unimportant [19]. This sense of mattering has been seen as a critical support for people during the COVID-19 pandemic [35], that there are true benefits from feeling that someone sees you as being important and valued.

In this regard, we extended past research that linked the new AMS measure with loneliness by showing that this association is not only present in university students (see [19]). It is also detectable among adults who have family members with serious mental illnesses. The findings align with the conclusion [18] that the anti-mattering construct has a particular focus on, and perhaps sensitivity to, feelings of being marginalized.

Examination of the qualitative responses gathered in the current study appears to mirror the social exclusion themes found in previous qualitative research [11,12], which are highlighted by the disturbing salience of the anti-mattering construct among the most stigmatized of our participants. Anti-mattering empirical findings are underscored by emotionally intense self-disclosures in participant responses about being shut out and cut off from other family members because of their relative with SMI. Feeling not listened to, feeling that what is being said is not important, and feeling like there is no opportunity to talk about their own experiences are troubling findings among these family members whose stigma reports seem poignantly linked to being ostracized even by those closest to them. This experience, for them, is too close for comfort.

There appears to be a dearth of interventions aimed at reducing stigma by association, according to a recent scoping review [36]. Future research should consider the implications of the current findings, particularly those that link societal stigma and marginalization with feelings of not mattering. Some suggested interventions have included transformative education, sharing, disclosure, social networking, and support, as well as public education, to correct misconceptions surrounding mental illness. The current findings highlight the importance of these interventions to address the specific need for heightened public mental health awareness surrounding stigma by association and the need to reach out with support to those marginalized individuals who are particularly affected by it.

One limitation of the current study relates to recruitment issues. Our sample was obtained by contacting local and nationally connected support organizations. The population of relatives of those with SMI has been particularly challenging to recruit for research participation, which is not surprising given the findings of social isolation. It is possible, and in fact quite likely, given the links of our participants to support organizations, that the prevalence of stigma by association is even higher, and the extent of loneliness and social isolation is underestimated in the broader population of family members. A further limitation is that the diagnoses of the relatives with mental illness were not independently verified; like other research in this area, we relied on family member self-reports.

## 5. Conclusions

The current study begins to address the gap in the existing family stigma literature by providing evidence of mental illness stigma by association, loneliness and social isolation in a North American sample. Through quantitative analyses, findings showed that the cohabiting family member group experienced higher levels of stigma by association and social isolation compared to the non-cohabiting group, though all participants experienced loneliness. The public health implications are that this is a marginalized group that is at serious risk for caregiver burnout, which would likely be exacerbated among caregivers who feel they are unappreciated and insignificant. The findings extend previous research, mainly restricted to qualitative studies, conducted in Scandinavia, Turkey, and Iran. In future studies, there is a need to examine societal interventions for reducing stigma by association as well as increasing the sense of mattering among family members, particularly those who live with a relative with SMI, because of the extent of their loneliness combined with heightened social isolation.

## Figures and Tables

**Table 1:** Participant Demographics.

Demographic	Cohabiting Family Member Group		Non-Cohabiting Family Member Group	
Participant	*n* = 81		*n* = 43	
Age in Years	*M* = 44.0 (*SD* = 17.1)		*M* = 53.8 (*SD* = 17.7)	
Female	*n* = 53	(65%)	*n* = 37	(86%)
Male	*n* = 27	(33%)	*n* = 6	(14%)
Non-binary	*n* = 1	(1%)	*n* = 0	(0%)
White/European	*n* = 54	(67%)	*n* = 32	(74%)
BIPOC	*n* = 27	(33%)	*n* = 11	(26%)
Mother/father	*n* = 36	(44%)	*n* = 25	(58%)
Sister/brother	*n* = 21	(26%)	*n* = 7	(16%)
Daughter/son	*n* = 11	(14%)	*n* = 3	(7%)
Spouse/partner	*n* = 11	(14%)	*n* = 0	(0%)
Other (e.g., aunt, cousin)	*n =* 2	(2%)	*n* = 8	(19%)

Note: BIPOC: Black, indigenous, and people of color.

**Table 2:** Demographics of Relatives with Mental Illness.

Demographic	Cohabiting Family Member Group		Non-Cohabiting Family Member Group	
Relative with Mental Illness	*n* = 81		*n* = 43	
Age in Years	*M* = 35.8 (*SD* = 13.66)		*M* = 40.6 (*SD* = 15.44)	
Female	*n* = 30	(37%)	*n* = 18	(42%)
Male	*n* = 48	(59%)	*n* = 24	(56%)
Non-binary	*n* = 3	(4%)	*n* = 1	(2%)
White/European	*n* = 56	(69%)	*n* = 30	(70%)
BIPOC	*n* = 25	(31%)	*n* = 13	(30%)
Schizophrenia	*n* = 32	(40%)	*n* = 17	(40%)
Other Psychotic Disorder	*n* = 34	(42%)	*n* = 13	(30%)
Bipolar Disorder	*n* = 22	(27%)	*n* = 16	(37%)
Major Depression	*n* = 14	(17%)	*n* = 10	(23%)
Other Mental Disorder	*n* = 14	(17%)	*n* = 11	(26%)

Note: The sum of the number and percentage of mental illnesses listed exceeds 124 and 100%, respectively, as each participant could report more than one condition. BIPOC: Black, indigenous, and people of color.

**Table 3:** Descriptive Statistics.

Measures	Total Sample		Cohabiting Family Member Group		Non-Cohabiting Family Member Group	
	*M*	*SD*	*M*	*SD*	*M*	*SD*
SAS	24.49	7.77	26.11	6.60	21.44	8.91
MSPSS SO	5.13	1.35	4.85	1.29	5.63	1.31
MSPSS FM	4.40	1.48	4.15	1.47	4.85	1.41
MSPSS FR	4.79	1.41	4.52	1.40	5.28	1.30
GMS	15.01	2.77	14.68	2.60	15.63	3.01
AMS	11.89	3.95	12.20	3.63	11.29	4.48
UCLA LS	46.16	14.02	47.22	14.09	44.19	13.83

Note: SAS: Stigma by Association Scale; MSPSS SO: Multidimensional Scales of Perceived Social Support from a Significant Other; MSPSS FM: Multidimensional Scales of Perceived Social Support from Family Members; MSPSS FR: Multidimensional Scales of Perceived Social Support from Friends; GMS: General Mattering Scale; AMS: Anti-mattering Scale; UCLA LS: UCLA Loneliness Scale.

**Table 4:** Pearson Correlation Coefficients (*r*) for all Participants and all Measures.

Measures	SAS	MSPSS SO	MSPSS FM	MSPSS FR	GMS	AMS
MSPSS SO	−0.09					
MSPSS FM	−0.24 **	0.54 ***				
MSPSS FR	−0.23 *	0.55 ***	0.52 ***			
GMS	−0.18 *	0.39 ***	0.30 ***	0.45 ***		
AMS	0.41 ***	−0.35 ***	−0.51 ***	−0.38 ***	−0.53 ***	
UCLA LS	0.34 ***	−0.53 ***	−0.45 ***	−0.48 ***	−0.44 ***	0.59 ***

* *p* < 0.05; ** *p* < 0.01; *** *p* < 0.001. Note: SAS: Stigma by Association Scale; MSPSS SO: Multidimensional Scales of Perceived Social Support from a Significant Other; MSPSS FM: Multidimensional Scales of Perceived Social Support from Family Members; MSPSS FR: Multidimensional Scales of Perceived Social Support from Friends; GMS: General Mattering Scale; AMS: Anti-mattering Scale; UCLA LS: UCLA Loneliness Scale.

## Data Availability

The datasets presented in this article are not readily available because the requesting source must be affiliated with an academic institution. Requests to access the datasets should be directed to the corresponding author: jgoldber@yorku.ca.

## Supplementary Materials

The following supporting information can be downloaded at: https://www.mdpi.com/article/10.3390/ijerph20065209/s1. The Stigma by Association Scale (SAS) adapted from Reference [21] is provided in the supplementary materials.

## References

[1] Rumas R., Shamblaw A.L., Jagtap S., Best M.W. (2021). Predictors and consequences of loneliness during the COVID-19 pandemic. Psychiatry Res..

[2] Holt-Lunstad J., Smith T.B., Baker M., Harris T., Stephenson D. (2015). Loneliness and social isolation as risk factors for mortality: A meta-analytic review. Perspect. Psychol. Sci..

[3] Best M.W., Bowie C.R. (2022). Social exclusion in psychotic disorders: An interactional processing model. Schizophr. Res..

[4] Wirsén E., Åkerlund S., Ingvarsdotter K., Hjärthag F., Östman M., Persson K. (2020). Burdens experienced and perceived needs of relatives of persons with SMI—A systematic meta-synthesis. J. Ment. Health.

[5] Kokurcan A., Özpolat A.G.Y., Gogus A.K. (2015). Burnout in caregivers of patients with schizophrenia. Turk. J. Med. Sci..

[6] Tabatabaee M., Yousefi Nooraie R., Mohammad Aghaei A., Rostam-Abadi Y., Ansari M., Sharifi S., Sharifi V. (2023). Loneliness in the presence of others: A mixed-method study of social networks of caregivers of patients with severe mental disorders. Int. J. Soc. Psychiatry.

[7] Phelan J.C., Bromet E.J., Link B.G. (1998). Psychiatric illness and family stigma. Schizophr. Bull..

[8] Struening E.L., Perlick D.A., Link B.G., Hellman F., Herman D., Sirey J.A. (2001). Stigma as a barrier to recovery: The extent to which caregivers believe most people devalue consumers and their families. Psychiatr. Serv..

[9] Corrigan P.W., Miller F.E. (2004). Shame, blame, and contamination: A review of the impact of mental illness stigma on family members. J. Ment. Health.

[10] Östman M. (2007). The burden experienced by relatives of those with a severe mental illness—Differences between those living with and those living apart from the patient. J. Psychiatr. Intensive Care.

[11] Van der Sanden R.L.M., Stutterheim S.E., Pryor J.B., Kok G., Bos A.E.R. (2014). Coping with stigma by association and family burden among family members of people with mental illness. J. Nerv. Ment. Dis..

[12] Van der Sanden R.L.M., Bos A.E.R., Stutterheim S.E., Pryor J.B., Kok G. (2015). Stigma by association among family members of people with a mental illness: A qualitative analysis. J. Community Appl. Soc. Psychol..

[13] Richard-Lepouriel H., Aubry J.M., Favre S. (2022). Is coping with sigma by association role-specific for different family members? A qualitative study with bipolar disorder patients’ relatives. Community Ment. Health J..

[14] Yin M., Li Z., Zhou C. (2020). Experience of stigma among family members of people with severe mental illness: A qualitative systematic review. Int. J. Ment. Health Nurs..

[15] Martens L., Addington J. (2001). The psychological wellbeing of family members of individuals with schizophrenia. Soc. Psychiatry Psychiatr. Epidemiol..

[16] Laidlaw T.M., Coverdale J.H., Fallon I.R., Fydd R.R. (2002). Caregiver’s stresses when living together or apart from patients with chronic schizophrenia. Community Ment. Health J..

[17] Elliott G., Kao S., Grant A.-M. (2004). Mattering: Empirical validation of a social-psychological concept. Self Identity.

[18] Flett G.L., Nepon T., Goldberg J.O., Rose A.L., Atkey S.K., Zaki-Azat J. (2022). The Anti-Mattering Scale: Development, psychometric properties, and associations with well-being and distress measures in adolescents and emerging adults. J. Psychoeduc. Assess..

[19] McComb S.E., Goldberg J.O., Flett G.L., Rose A.L. (2020). The double jeopardy of feeling lonely and unimportant: State and trait loneliness and feelings and fears of not mattering. Front. Psychol..

[20] Taylor J., Turner R.J. (2001). A longitudinal study of the role and significance of mattering to others for depressive symptoms. J. Health Soc. Behav..

[21] Tessler R., Gamache G. (1993). Evaluating Family Experiences with Severe Mental Illness.

[22] Zimet G.D., Dahlem N.W., Zimet S.G., Farley G.K. (1988). The multidimensional scale of perceived social support. J. Personal. Assess..

[23] Marcus F.M., Rosenberg M. Mattering: Its measurement and significance in everyday life. Proceedings of the 57th Annual Eastern Sociological Society Meeting.

[24] Flett G.L. (2018). The Psychology of Mattering: Understanding the Human Need to Be Significant.

[25] Russell D., Peplau L.A., Ferguson M.L. (1978). Developing a measure of loneliness. J. Personal. Assess..

[26] R Core Team (2022). R: A Language and Environment for Statistical Computing.

[27] Burnard P. (1991). A method of analysing interview transcripts in qualitative research. Nurse Educ. Today.

[28] Van der Sanden R.L.M., Bos A.E.R., Stutterheim S.E., Pryor J.B., Kok G. (2013). Experiences of stigma by association among family members of people with mental illness. Rehabil. Psychol..

[29] Van der Sanden R.L.M., Pryor J.B., Stutterheim S.E., Kok G., Bos A.E.R. (2016). Stigma by association and family burden among family members of people with mental illness: The mediating role of coping. Soc. Psychiatry Psychiatr. Epidemiol..

[30] Wahl O.F., Harman C.R. (1989). Family views of stigma. Schizophr. Bull..

[31] Ostman M., Kjellin L. (2002). Stigma by association: Psychological factors in relatives of people with mental illness. Br. J. Psychiatry.

[32] Zimet G.D., Powell S.S., Farley G.K., Werkman S., Berkoff K.A. (1990). Psychometric characteristics of the Multidimensional Scale of Perceived Social Support. J. Personal. Assess..

[33] Li H. (2023). Maternal-Infant Attachment and its Relationships with Postpartum Depression, Anxiety, Affective Instability, Stress, and Social Support in a Canadian Community Sample. Psychiatr. Q..

[34] Stain H.J., Galletly C.A., Clark S., Wilson J., Killen E.A., Anthes L., Harvey C. (2012). Understanding the social costs of psychosis: The experience of adults affected by psychosis identified within the second Australian National Survey of Psychosis. Aust. N. Z. J. Psychiatry.

[35] Flett G.L., Zangeneh M. (2020). Mattering as a vital support for people during the COVID-19 pandemic: The benefits of feeling and knowing that someone cares during times of crisis. J. Concurr. Disord..

[36] Adu J., Oudshoorn A., Anderson K., Marshall C.A., Stuart H., Stanley M. (2021). Policies and interventions to reduce familial mental illness stigma: A scoping review of empirical literature. Issues Ment. Health Nurs..

