# Supplementary material for: Too Close for Comfort: Stigma by Association in Family Members Who Live with Relatives with Mental Illness
Source: Int J Environ Res Public Health. 2023 Mar 16;20(6):5209. doi: 10.3390/ijerph20065209 (PMC10049681; doi:10.3390/ijerph20065209)
Supplement: Supplementary file 1 [file ijerph-20-05209-s001.zip › ijerph-2249801-SI.pdf]

The Stigma by Association Scale

(SAS; adapted from Tessler & Gamach, 1993)

INSTRUCTIONS: People who are related to an individual with serious mental illness may at times find that they too are stigmatized by others due to their association with their family member who has mental health issues. Please use the 5-point scale to rate the degree to which you have experienced each item. Choose the number that best corresponds with your opinion.

1 = Never, 2 = Rarely, 3 = Sometimes, 4 = Often, 5 = Always

1. I have worried whether people would find out about my relative's mental illness
2. I have worried that my neighbours would treat me differently because of my relative's mental illness.
3. I have felt the need to hide my relative's mental illness.
4. I have kept my relative's mental illness a secret.
5. I have worried that friends and neighbours would avoid me after they found out my relative's mental illness.
6. I did not see some of my friends and neighbours as often as I did before because of my relative's mental illness.
7. I avoided going to large parties or social events with my relative because of their mental illness.
8. I worried that even my best friends would treat me differently because of my relative's mental

   illness.
9. I felt ashamed or embarrassed about my relative's mental illness.

Tessler, R.; Gamache, G. *Evaluating family experiences with severe mental illness*; Human Services Research Institute: Cambridge, MA, USA, 1993; p. 87.
